# Culturally sensitive adaptation of the concept of relational communication therapy as a support to language development: An exploratory study in collaboration with a Tanzanian orphanage

**DOI:** 10.4102/sajcd.v63i1.166

**Published:** 2016-11-07

**Authors:** Ulrike Schütte

**Affiliations:** 1 Department of Speech and Language Pedagogy and Therapy, Leibniz University of Hannover, Germany

## Abstract

**Background:**

Orphans and other vulnerable children (OVC) who grow up in institutional care often show communication and language problems. The caregivers lack training, and there are few language didactics programmes aimed at supporting communication and language development in OVC in institutional care in Tanzania.

**Objectives:**

The purpose of the study was to adapt the German concept of relational communication therapy (RCT) as a support to language development in a Tanzanian early childhood education context in a culturally sensitive way. Following the adaptation of the concept, a training programme for Tanzanian caregiver students was developed to compare their competencies in language didactics before and after training.

**Methods:**

A convergent mixed methods design was used to examine changes following training in 12 participating caregiver students in a Tanzanian orphanage. The competencies in relational language didactics were assessed by a self-developed test and video recordings before and after intervention. Based on the results, we drew conclusions regarding necessary modifications to the training modules and to the concept of RCT.

**Results:**

The relational didactics competencies of the caregiver students improved significantly following their training. A detailed analysis of the four training modules showed that the improvement in relational didactics competencies varied depending on the topic and the teacher.

**Conclusion:**

The results provide essential hints for the professionalisation of caregivers and for using the concept of RCT for OVC in institutional care in Tanzania. Training programmes and concepts should not just be transferred across different cultures, disciplines and settings; they must be adapted to the specific cultural setting.

## Introduction

According to current figures, there are an estimated 3 million – and counting – orphans living in Tanzania (UNICEF, 2013). As a result, extended families and community-based care programmes are overburdened (Subbarao & Coury, 2004), and orphanages and other institutional care facilities are catering for many orphans and other vulnerable children (OVC). International studies have provided documentary evidence that children who grow up in institutional care show developmental disorders – especially if the children are placed in institutional care at a very young age (Hermenau, Hecker, Elbert & Ruf-Leuschner, 2014; Smyke, Zeanah, Fox, Nelson & Guthrie, 2010). Children who have been institutionalised often show delayed physical and cognitive development (Dobrova-Krol, IJzendoorn, Bakermans-Kranenburg & Juffer, 2010; Merz & McCall, 2010) and suffer from social, behavioural and psychological problems (Hermenau *et al*., 2011; Oliveira *et al*., 2012). Furthermore, OVC are at risk of delayed speech and language development (Glennen, 2002; Levin & Haines, 2007; St. Petersburg USA Orphanage Research Team, 2008).

The reasons are manifold: on the one hand, caregivers lack training in areas such as child-rearing – especially in relation to children who have developmental problems (Muhamedrahimov, Palmov, Nikiforova, Groark & McCall, 2004; Naqshbandi, Mudasir, Rashmi & Hassan, 2012). Instead, the caregivers focus on medical care and routine daily caring. On the other hand, there is a lack of language didactics concepts for encouraging communication and language development in OVC (Hermenau *et al*., 2014).

This is the point of departure for the present study. Using a culturally adapted version of the concept of relational communication therapy (RCT) as a support to language development (Lüdtke, 2012b) in a Tanzanian early childhood education context, a training programme for the caregiver students at a Tanzanian orphanage was developed, implemented and evaluated in a cooperative venture between Sebastian Kolowa Memorial University in Tanzania and Leibniz University Hannover in Germany (Schütte *et al*., 2016). By capturing the theoretical relational competencies (TRC), the methodological relational competencies (MRC) and the dialogical relational competencies (DRC) (Lüdtke, 2010) of the caregiver students, I was able to reach certain conclusions about the influence of the training and as a consequence about the concept and the culturally sensitive way it was adapted. The changes in the children were not assessed.

Research with and ‘on’ orphans is a highly sensitive topic. Carrying out this type of research in one of Germany’s former colonies and in a church-run institution implies the need for a culturally sensitive approach. Cultural sensitivity covers everything from the establishment of a mutually satisfactory relationship of trust between the parties (Westby & Hwa-Froelich, 2003) to a commitment to the results benefiting all research participants. Numerous studies throughout the world showing a preference for research *with* rather than *on* people support this approach (Davis & Reid, 1999; Koch, Selim & Kralik, 2002). Following the same line of thinking, it is also argued that speech therapy models and training concepts cannot be transferred between different cultural contexts and countries without culturally sensitive adaptation (Barrett & Marshall, 2013). Therefore, the concept and resulting training programme was developed, implemented and evaluated on the principles of the participatory action research approach (Reason & Bradbury, 2009).

### Culturally sensitive adaptation of the concept of relational communication therapy as a support to language development for vulnerable children in early institutional care

In this study, cultural sensitivity and participatory action research (PAR) were closely interdependent (Reason & Bradbury, 2009). PAR implies a form of research that is carried out with the involvement of all protagonists, addresses actual problems, discusses and reflects on the results and also models follow-up actions in discussion with all concerned parties. As such, PAR is described as a cycle in which reflexive and active parts mutually influence and enforce one another (Reason & Bradbury, 2009). In particular, the emphasis on an openness towards intersubjective dialogue is of great importance. PAR implies the shaping of community relations between protagonists and the opening of new areas of communication in which dialogue and development may progress (Reason & Bradbury, 2009). Dialogical relationships and intersubjective construction of meaning therefore serve as key theoretical concepts at the different levels described in this article: (1) the innate intersubjectivity theory (Trevarthen & Aitken, 2001) with regard to the caregiver–child dialogue, (2) in the relational theory of communication and language acquisition (Lüdtke, 2012a), (3) in approaches to foster children who have developmental communication and language disorders (Frank & Lüdtke, 2012) as well as in (4) the didactical orientation of the video interaction guidance (VIG) approach (Kennedy, Landor & Todd, 2011). In particular, the ethical dimension as well as the tacit power differences in international development cooperation require an obligatory focus on the global common good (Walker & Early, 2010). This shows that the principles of PAR are fundamentally bound up with culturally sensitive work. Within the framework of this study, the concept of RCT plays a fundamental role and was adapted in a culturally sensitive manner to the early childhood education context with regard to working with OVC in Tanzania.

**FIGURE 1:**
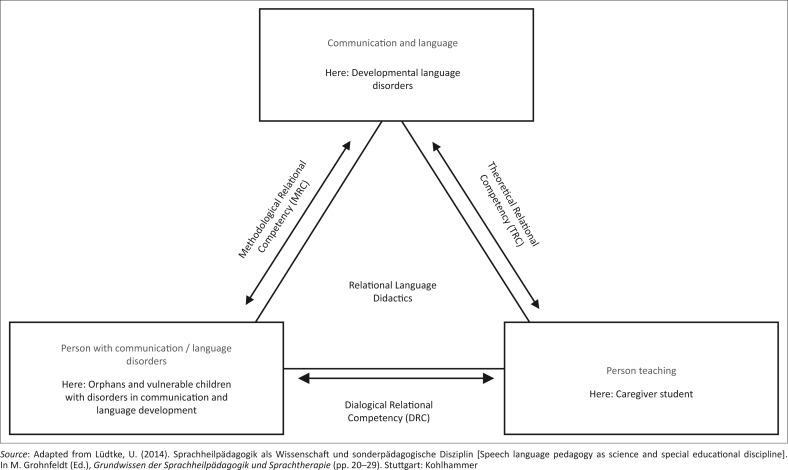
Adapted, culturally sensitive relational language didactics triangle.

**FIGURE 2:**
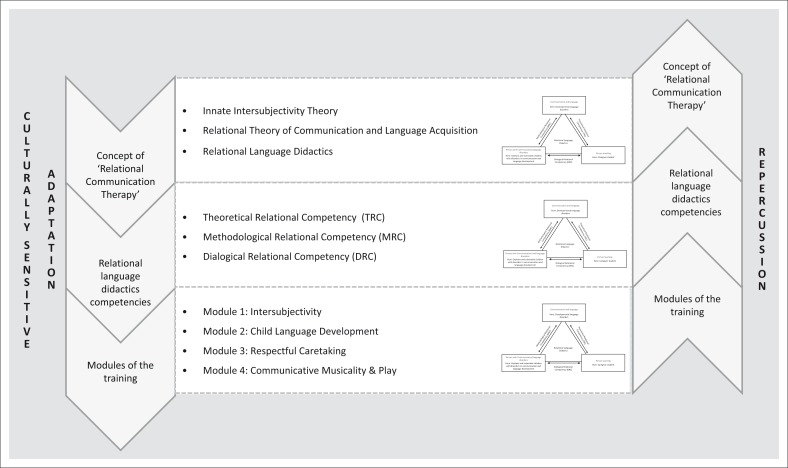
Interacting theoretical levels.

It is not the aim of this article to describe the concept of RCT, which originally focused on mother–child interactions, in both the German and Tanzanian contexts. Instead, I simply discuss the adapted Tanzanian model with the modifications made for this special cultural context. These modifications are marked in the text and in Figure 1 with the word ‘here’. The derived competencies and modules of the concept in particular have been adapted to be culturally sensitive to the Tanzanian orphanage setting (see Figure 1 and Table 1) and influence one another (see Figure 2).

**TABLE 1:** Adapted, culturally sensitive training modules.

Module	General module description	Selected topics
1: Innate intersubjectivity	Assumes that the child (here: OVC) expects an emotionally communicating person (here: caregiver student) right after birth and that the child has an inborn need to communicate (Trevarthen & Aitken, 2001). It also includes forms of deviating behaviour that could occur in orphanages when the opportunity for mutual communication situations is limited.	Building up communication, e.g. signs that a newborn is ready to interact. Abilities of the newborn, e.g. a newborn can see, hear, smell and feel another person immediately after birth. Communicative needs, e.g. interpreting a child’s cry. Disturbed communication, e.g. signs of developmental problems.
2: Child language development	Concerns the child’s communication and language development in intersubjective relationships (here: caregiver student and OVC relationship). Relational emotions are seen as the basis for communication and language development (Lüdtke, 2012b).	Development of subjective and intersubjective motives. Different time frames and the appropriate way of behaving in communication, e.g. responding to the child’s pointing at an object by naming it. Different time frames and the appropriate stage of development.
3: Respectful caretaking	Relates to an approach developed by Emmi Pikler (founder of an orphanage in Hungary) based on respect for babies as human beings. Children are considered as active participants in their care from birth, never passive objects or recipients of care (Pikler & Tardos, 2014).	Building up an emotionally meaningful relationship, e.g. involving attention, body contact, etc. The importance of an emotionally meaningful relationship for communication and language development. Abilities of children in the caring activities, e.g. assistance in dressing. Verbal and non-verbal accompaniment, e.g. accompanying verbalisation of actions.
4: Communicative musicality and play	Related to African musical culture, this module emphasises the ‘natural’ communicative musicality aspect that children are born with. This means that rhythmic sounds and melodies are recognised before and memorised after birth (Trevarthen, 2008). Furthermore, the importance of play in development is focused on.	Different types of songs to regulate the child’s emotions, e.g. play songs. Different ways of expressing emotions, e.g. rhythmical movements. Different ways of exchanging rhythms with the child, e.g. mirroring rhythm. Importance of play in development, e.g. for communication and language development.

The focus of the present study was on delayed communication and language development in OVC. Levin and Haines (2007) show that every child in the orphanage they studied in South Africa had speech and language difficulties. One of the contributory reasons for this is a lack of response from the caregivers. They speak rarely or not at all to the children. Interactions usually happen in a directive and not a child-oriented manner. Furthermore, caregivers do not establish eye contact and do not respond to the communicative attempts of the children (Levin & Haines). The concept of RCT adapted in a culturally sensitive manner addresses the root of this problem.

‘Language didactics’ is defined as the professional organisation of specific communication and language teaching and learning processes (Lüdtke & Stitzinger, 2015). As such, the intuitive behaviour of parents interacting with their children (Papoušek & Papoušek, 1983) is taken up by professionals and didactically presented to the participating caregiver students. The concept is based on the following three elements.

#### Innate intersubjectivity theory

Even newborn babies have an intrinsic motive to make contact with somebody meaningful. This is understood to be a precondition for an individual’s ‘cultural learning’. Trevarthen and Aitken (2001) describe this as ‘innate intersubjectivity’. The need for an intersubjective exchange of emotions is seen as the basis of social learning and brain development and the closely related cognitive learning progress (Lüdtke, 2012b). Even newborn babies can mirror emotions (Trevarthen & Aitken, 2001) and undertake or even initiate emotional interaction (Nagy & Molnar, 2004).

#### Relational theory of communication and language acquisition

The relational theory of communication and language acquisition, which refers to innate intersubjectivity theory, sees communication and language development as a co-constructing process in which meaning is developed in intersubjective and emotionally meaningful situations (Lüdtke, 2012b). The learning child (here: OVC) needs an emotionally responsive communicating other (here: caregiver student) for successful language development. Relational emotions and their reciprocal mirroring are crucial for communication and language development (Lüdtke, 2010).

#### Relational language didactics

Relational language didactics is based on the principles of the relational theory of language acquisition and constructivist pedagogy and didactics (Lüdtke, 2012a). Constructivist pedagogy and didactics assumes that learning cannot be reduced to the reproduction of existing knowledge but needs to be understood as interplay between internal construction and instruction from the outside (Reich, 2004). In this sense language-learning is an active acquisition process in which the person teaching (here: caregiver student) plays an important role (Reich, 2004). Relational language didactics integrates the two elements referred to above to describe the development of severe communication and language disorders. Methodologically, this implies that professionals need to account for the function of emotions in communication and language development (Lüdtke, 2010). That in turn means that, for example, a child (here: OVC) needs an emotionally meaningful communicating other (here: caregiver student) if he or she is to develop an awareness of the meaning of signs in interactions and to use these emotionally meaningful interactions as motivation for further learning.

Based on the culturally adapted concept, the project team operationalised competencies in relational language didactics for a training programme for Tanzanian caregiver students.

### Relational language didactics competencies as adapted in a culturally sensitive manner

‘Competency’ is a wide-ranging term and can be understood from different perspectives and in a different way depending on the academic discipline. In our particular language didactics context, I refer to the relational language didactics model proposed by Lüdtke (2014, 2012a) (see Figure 1). It assumes an interdependent triangular set of referentials in the language didactics teaching and/or learning process (Lüdtke, 2010):

- the *person with communication* and/or *language disorders* (here: OVC with communication and language development disorders)
- the *person* *teaching* (here: caregiver student) and
- *communication and language and their impairments* (here: developmental language disorders).

The aim of the training is for the caregiver student to acquire the competencies to successfully combine two of the components in the relational language didactics triangle (Lüdtke, 2010):

- The TRC covers profound interdisciplinary knowledge of how therapeutic didactic decisions connect to the emotive linguistic specifics of the intersubjective language-learning process (here: knowledge about the intersubjective language-learning process between the caregiver student and OVC) (e.g. knowledge about the communicative abilities of a newborn child).
- The MRC covers the ability to apply acquired techniques in the given language-learning contexts to the person with communication and language disorders (here: OVC with disorders in communication and language development). This includes the design of a suitable environment for the developmental stage of the child’s communication abilities (e.g. establish eye contact).
- The DRC covers the ability of the person teaching (here: caregiver student) to make and to maintain an appropriate dialogical connection to the client (here: OVC) (e.g. to recognise and respond positively emotionally to the communicative attempts of the OVC).

Taking the culturally sensitive adaptation of RCT as a basis, four modules suggest themselves, reflecting the content-related and methodological specifics of the Tanzanian orphanage setting (see Table 1).

The levels described above overlap, complement and influence one another (see Figure 2). This also accounts for possible changes in the concept of RCT that may occur as a result of the training in relational language didactics competencies.

## Research method and design

### Setting

The empirical study was conducted in an orphanage in rural Tanzania, affiliated to the Evangelical-Lutheran church. The institution was under missionary influence for many years but is now becoming an increasingly inclusive institution with a focus on preventive thinking. At the time of the study, the orphanage had 2 managers, 8 permanent employees, 25 apprentices and 32 children ranging from newborn babies to 3-year-olds. The orphanage obtained its funding through donations and the sale of agricultural produce.

### Participants

Twelve caregiver students at this Tanzanian orphanage took part in the study (mean age = 22 years; age range = 21–28 years; all female). They belonged to five different ethnic groups (Ndembwike, 2006) – as a result, 10 out of the 12 participants grew up speaking multiple languages. All of them were in their second and final year of apprenticeship. The caregiver students received only limited training with regard to childcare. The emphasis of their apprenticeship was generally on agriculture, housekeeping and business matters. The caregiver students had no final secondary-school qualification, which significantly complicated their vocational integration. Therefore the training was specifically designed in an easy-access way using non-academic Swahili instructions.

### Research questions

The aim of this study was to adapt the concept of RCT to a culturally sensitive training programme for caregiver students in a Tanzanian orphanage. Changes in communicative and language behaviour between the caregiver students and the children were captured using a pre-post design. Pre-post design is based on the principle that pedagogical–therapeutic competencies are not fixed but evolve progressively and change through internal or external aspects (Kaufhold, 2006). As a result, the two main research questions were the following: (1) To what extent do the theoretical, methodological and dialogical relational competencies (TRC, MRC and DRC) of the caregiver students change as a result of training? (2) Which module-specific differences in the theoretical, methodological and dialogical relational competencies (TRC, MRC and DRC) can be found in the caregiver students after taking part in training?

Both research questions are discussed here with regard to the TRC and MRC perspectives of the adapted, culturally sensitive, relational language didactics triangle. The description of the changes in DRC can be found in future publications.

### Research design

**FIGURE 3:**
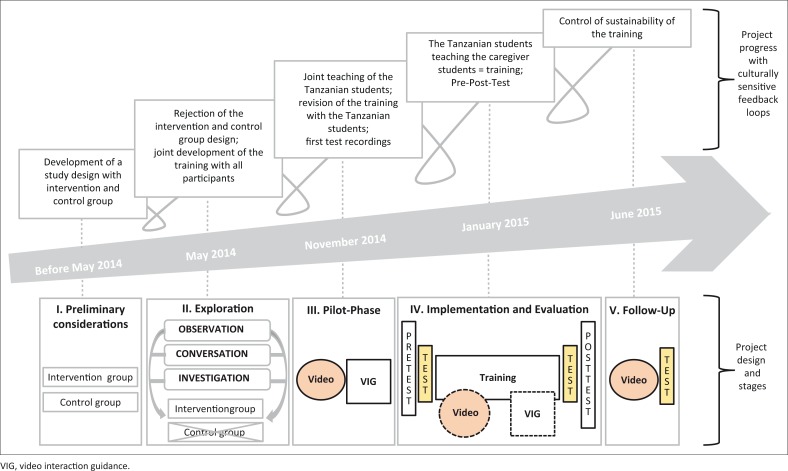
Culturally sensitive study design.

The culturally sensitive design (see Figure 3) was carried out according to the standards of focused ethnography. This implies shorter field research, focusing on situations in which communicative activities as well as specific forms of personal interaction could be studied (Tuma, Schnettler & Knoblauch, 2013). The culturally sensitive design was characterised by an intensive and equal collaboration between all German and Tanzanian scientists and students as well as the orphanage and the church. The collaborative relationship strengthened over the course of the project. Figure 3 illustrates these aspects through culturally sensitive feedback loops:

- At the *preliminary considerations* stage, we organised joint study design workshops. We planned to capture the effects of the training with a quantitative control and intervention group design (Punch, 2014).
- This phase was followed by the *exploration phase* to understand the needs of the study participants. As a first step, the management of the orphanage was involved in setting the thematic framework of the training programme and discussing the local situation. Participant observation was used at this stage to deepen understanding of cultural specifics from the inside (Friebertshäuser, 2010). During conversations with the orphanage management, it became clear that the biggest need was to gain theoretical and practical knowledge in the field of communication and language promotion, with a focus on establishing an emotionally responsive dialogue between caregiver students and children. Cross-cultural research into the literature relating to the training topics rounded off this phase of exploration. As a result of the exploration process, we had to abandon the planned control and intervention group design due to specific local circumstances (e.g. the high turnover of caregiver students and cohabitation in the orphanage). This example also illustrates the constant movement and development of the methodology as it evolved as part of a mutual process that included all those involved.
- During the *pilot phase*, the German and Tanzanian project partners worked together with Tanzanian students from Sebastian Kolowa Memorial University on defining the topics for the training programme. Firstly, the students were familiarised with the theoretical content of the four modules and reflected on the adaptability of the content to the Tanzanian cultural and institutional context. After that, the detailed goal descriptions for TRC, MRC and DRC were operationalised as concrete competencies (see Table 2). In addition, the caregiver students recorded the first videos of their interaction with the children. Based on this, they received their first video feedback via VIG (Kennedy *et al*., 2011).
- Following this, *implementation and evaluation* of the training took place. The caregiver students participated in a 4-week training programme (four modules with three teaching units) on the topics of innate intersubjectivity, child language development, respectful caretaking and communicative musicality and play (see Table 1). The Tanzanian students gave the training in Swahili to ensure cultural and linguistic proximity among caregiver students. The German team took a supervisory role. Over the course of the study, both caregiver students and Tanzanian students were recorded during interactions with the children or during instructions. Both groups received feedback and supervision using VIG. The training was rounded off with pre- and post-testing, which consisted of two components. Firstly, a self-developed informal competence test was administered in Swahili to capture TRC and MRC. The second component consisted of video recordings, which were used to capture DRC before and after the training. Because the capturing of DRC requires direct interaction with an emotional meaningful other, it was thus not part of the questionnaire.
- Four months after the training, long-term data were collected in the *follow-up*. It consisted of the informal competency test to capture TRC and MRC as well as video recordings to capture DRC. The follow-up served as a control measure with regard to the sustainability of the training and the effects of learning. The results of the follow-up are described and discussed as part of the dissertation project of my Tanzanian colleague J. Semkiwa (work in progress).

**TABLE 2:** Extract from detailed goal descriptions and subsequent questions from the informal competency test for modules 1 and 2.

Module	Competency	Aim	Question
1	TRC	The caregiver knows that the cry of a child symbolises a demand to have needs fulfilled.	What can the cry of a newborn mean? The newborn is hungry. The newborn has a stomach ache. The newborn needs somebody to pick him or her up. It does not mean anything.
	MRC	The caregiver tries out different ways of soothing the child (e.g. feeds the child if he or she is hungry, communicates with the child, etc.).	Please imagine the following situation: A caregiver is washing a newborn baby. Suddenly the newborn starts to cry. Which of the following caregivers reacts well? Caregiver 1 stops washing the newborn. She soothes the newborn. Caregiver 2 continues cleaning the child. The newborn will calm down on its own. Caregiver 3 stops washing the child. After some minutes the newborn starts to cry less. The caregiver goes back to washing the child. Caregiver 4 says: ‘Stop crying!’ Then she continues washing the child.
2	TRC	The caregiver knows that a child at the age of 13–24 months points at objects to create joint interest, produces sounds similar to the surrounding language, combines gestures and vocalisations, and starts to use first words and that afterwards he or she expands his or her vocabulary.	Which linguistic behaviour is typical for a child at the age of 13–24 months? The child points at objects. The child combines gestures with vocalisations. The child starts to speak his or her first words. The child produces sounds similar to Swahili.
	MRC	The caregiver provides cooperative situations: e.g. situations in which the child has a chance to point at objects; situations in which the child has the chance to combine gestures and vocalisations; and situations of joint cooperation designed to encourage first words.	Which of the following caregivers support the child’s communication development well? The child is 13–24 months old. Caregiver 1 walks around with the child. She shows the child different objects from the world outside. Caregiver 2 gives the child toys. Caregiver 3 plays together with the child and a teddy. Caregiver 4 leaves the child in bed.

TRC, theoretical relational competency; MRC, methodological relational competency.

#### Instruments

The present study is based on the requirements of summative evaluation (Westermann, 2002). It assessed the competencies of the participating caregiver students both before and after training. It was analysed so as to reveal if, and to what extent, the TRC, MRC and DRC of the caregiver students changed after training. Because of the lack of a control group and the small sample size, it cannot be demonstrated reliably that there is a causal connection between the changes in the competencies and the training. For that reason, the data before and after training can be assessed but not necessarily causally linked. Moreover, the results can be related to the examined group *only* and *cannot* be used to make generalisations.

### Mixed methods design

**FIGURE 4:**
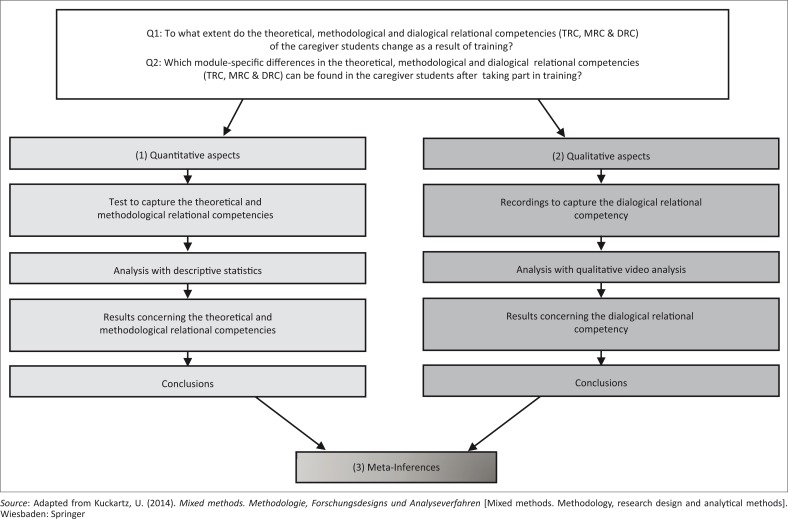
Detailed schema of the convergent design.

The complete study consisted of three substudies and is based on a convergent design (see Figure 4). It involved the separate collection and analysis of (1) quantitative and (2) qualitative data with the intention of (3) merging the results of both strands at the end of the research to obtain meta-inferences (Creswell, 2015).

This procedure was used for both the pre- and the post-test. The meta-inferences of both points of data collection can then be correlated. I will concentrate here on the (1) quantitative inferences of the pre- and post-test (see Figure 4 left strand). The (2) qualitative results and the (3) merging of the results will be discussed in future publications.

#### Informal competency test

The informal competency test was developed at the request of the orphanage managers to capture TRC and MRC before and after training. It consisted of 27 questions (with multiple possible answers) focusing on the four topics included in the training (see Table 1). These questions captured either TRC or MRC. Each detailed goal description was operationalised in one question (see Table 2). Completing the test took approximately 30 min.

The informal competency test fulfilled the main psychometric criteria (Bühner, 2006) as mentioned below.

##### Objectivity

The test was completed by each caregiver student independently at the same time. Additionally, the test contained only closed-ended multiple-choice questions, with the result that the interpretation was unambiguous due to the objectivity.

##### Reliability

The reliability of the test was analysed and checked using SPSS statistical analysis software. Items that did not correlate well with the total scale were removed from the test. This led to a total of 27 questions instead of the original 38. Consequently, the informal competency test showed acceptable internal consistency (Cronbach’s alpha *α* = 0.75) (Field, 2009).

##### Validity

In order to increase the validity of the test, the phrasing of the questions followed a simple yet precise format to keep questions strictly related to the subject in hand and to guarantee that the instrument measured the intended topic.

A Swahili native speaker from the research team translated the questions from English into Swahili, using easy non-academic language where possible. A second member of the team approved the phrasing.

#### Data analysis

The results of the tests were generated using SPSS to obtain descriptive statistics and Spearman correlations, as well as to check statistical significance from the first time of measurement to the second one (t1 to t2) by the non-parametric Wilcoxon signed-rank test. Because the questions were operationalised to show either TRC or MRC transfer, I was able to draw conclusions about each caregiver student’s competency in both areas. In this way I was also able to assess, for example, whether a caregiver student had comprehensive knowledge about innate intersubjectivity but had not yet put it into practice in methodological procedures.

## Results

To answer the first research question (Q1) concerning the shift of TRC and MRC post-training, an overview of the overall development of the 12 caregiver students as well as the shift in TRC and MRC pre- and post-test follows. TRC was not significantly related to MRC (*rs* = 0.11, *p* = 0.735), and thus these areas are analysed separately.

**FIGURE 5:**
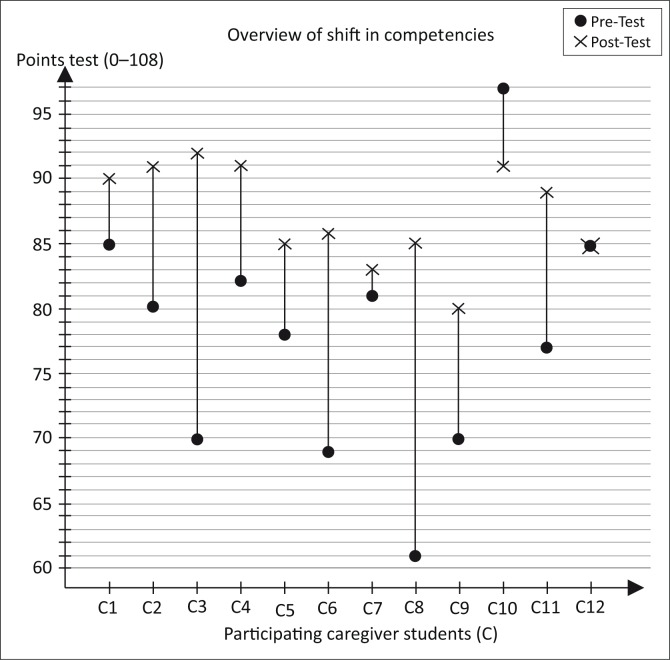
Overview of shift in competencies (TRC and MRC) of the 12 caregiver students (C1–C12).

Ten out of the twelve caregiver students increased the number of correctly answered questions in the informal competency post-test (see Figure 5). In the case of one student (C10), the number of correctly answered questions decreased, whereas one student showed no difference before and after training (C12).

Because of the small sample size, the appropriate measure of central tendency was the median, and the appropriate statistical test was the Wilcoxon signed-rank test. Table 3 shows that the median of the post-test (t2) was higher than that of the pre-test (t1) (in all three examined units). The interquartile range (IQR) describes the mean distribution and is used for the present study as an appropriate statistical dispersion. In all of the three examined units the IQR decreased – therefore the dispersion decreased (see Table 3).

**TABLE 3:** Results in total and by competency.

Results	t1				t2				
	Empirical range	Md	Md range	IQR	Empirical range	Md	Md range	IQR	*α*
Points total	61–97	79.0	0–108	14.25	80–92	87.5	0–108	6.0	0.75
TRC total	41–63	53.0	0–68	10.25	50–60	57.5	0–68	3.5	0.75
MRC total	17–35	26.0	0–40	6.50	25–34	30.5	0–40	4.5	0.71

TRC, theoretical relational competencies; MRC, methodological relational competencies; Md, median; IQR, interquartile range; t1, pre-test; t2, post-test; α, Cronbach’s alpha.

Table 4 illustrates a significant difference between pre- and post-test for both the total results and the results of TRC and MRC. The effect size *r* is ≥ 0.7 for all three examined units. This signifies a strong effect (Field, 2009).

**TABLE 4:** Results of the Wilcoxon signed-rank test: Theoretical relational competency and methodological relational competency of all modules in total and of each module separately.

Test statistic	Variable	*N*										
		All modules			Innate intersubjectivity		Child language development		Respectful caretaking		Communicative musicality and play	
		Total	TRC	MRC	TRC	MRC	TRC	MRC	TRC	MRC	TRC	MRC
Post-test – Pre-test	Z	-2.67	-2.43	-2.59	-2.83	-1.92	-0.50	-1.87	-0.64	-2.21	-2.26	-2.05
	Asymp. Sig. (2-tailed)	0.008	0.015	0.009	0.005	0.055	0.620	0.062	0.521	0.027	0.024	0.041
	Effect size r	0.77	0.70	0.74	0.81	0.55	0.14	0.53	0.18	0.63	0.65	0.59
Ranks	Negative ranks	1^a^	2^a^	3^a^	0^a^	3^a^	3^a^	2^a^	4^a^	1^a^	1^a^	3^a^
	Positive ranks	10^b^	10^b^	9^b^	10^b^	8^b^	5^b^	6^b^	4^b^	9^b^	9^b^	8^b^
	Ties	1^c^	0^c^	0^c^	2^c^	1^c^	4^c^	4^c^	4^c^	2^c^	2^c^	1^c^
**Total**	**-**	**12**	**12**	**12**	**12**	**12**	**12**	**12**	**12**	**12**	**12**	**12**

a post-test < pre-test;

b post-test > pre-test;

c post-test = pre-test.

TRC, theoretical relational competency; MRC, methodological relational competency.

As a second step I sketch the module-specific differences in TRC and MRC in the caregiver students following the training in order to answer research question Q2. The Spearman correlation analysis draws the following picture: innate intersubjectivity TRC and MRC (*rs* = 0.06, *p* = 0.853), child language development TRC and MRC (*rs* = 0.45, *p* = 0.139), respectful caretaking TRC and MRC (*rs* = 0.50, *p* = 0.097) and communicative musicality and play TRC and MRC (*rs* = 0.38, *p* = 0.220). There are no relevant correlations, so all areas will be analysed separately.

The module-specific analysis showed that the median increased from pre- to post-test for the topics of innate intersubjectivity, child language development, and communicative musicality and play in TRC and MRC. For the topic of respectful caretaking, the median decreased in TRC but increased in MRC. The IQR decreased for all examined units – therefore the dispersion decreased. Only for the topic of child language development did the IQR increase in MRC (see Table 5).

**TABLE 5:** Results of the module-specific analysis of the training topics.

Variable	Competency	t1				t2			
		Empirical range	Md	Md range	IQR	Empirical range	Md	Md range	IQR
Innate intersubjectivity	TRC	3–12	9.5	0–12	1.75	10–12	11.0	0–12	1.00
	MRC	8–12	9.5	0–12	1.75	9–12	11.0	0–12	1.00
Child language development	TRC	16–23	19.5	0–24	3.75	15–22	20.0	0–24	2.75
	MRC	1–7	5.5	0–8	2.00	3–8	6.0	0–8	3.00
Respectful caretaking	TRC	6–11	9.5	0–12	2.75	7–11	9.0	0–12	1.00
	MRC	8–17	12.0	0–20	4.75	10–16	15.0	0–20	2.50
Communicative musicality and play	TRC	15–24	18.0	0–24	3.75	15–22	20.0	0–24	1.75
	MRC	2–8	4.0	0–8	2.75	3–7	6.0	0–8	1.75

TRC, theoretical relational competencies; MRC, methodological relational competencies; t1, pre-test; t2, post-test; Md, median; IQR, interquartile range.

The differences between pre- and post-test were examined by means of inferential statistics (see Table 4).

It is evident that TRC in the innate intersubjectivity module differed significantly before and after the training. The effect size *r* = 0.81 indicates that it was a strong effect (Field, 2009). MRC showed no significant difference. In the child language development module neither TRC nor MRC showed a significant difference between pre- and post-test. For the respectful caretaking topic it can be concluded that TRC showed no significant difference between pre- and post-test. By contrast, MRC showed a significant difference before and after the training. The effect size *r* = 0.63 illustrates that it was a strong effect (Field, 2009). In the communicative musicality and play module, TRC and MRC indicated a significant difference between pre- and post-test. The effect size *r* = 0.65/0.59 shows that it was a strong effect (Field, 2009) (see Table 4).

## Ethical considerations

The study was conducted in an ethically responsible manner and with the clear consent and acceptance of those involved. Participants had the right to withdraw their written informed consent at any time during the research process. The ethics committee at Leibniz University of Hannover inspected the application in-depth and approved it. The Tanzanian team obtained research clearance through the Tanzanian Commission for Science and Technology ethics committee. In addition, the head of the church and the managers of the orphanage consented to the study in writing and were involved in all stages of the research.

## Discussion

Owing to the lack of language didactics concepts for OVC in institutional care in Tanzania, the current study focused on a culturally sensitive adaptation of the German concept of RCT. Based on the adapted model, a training programme for the caregiver students at a Tanzanian orphanage was developed, implemented and evaluated. The first research question (Q1) addressed the shift in TRC and MRC after training as illustrated by the overall development of the 12 caregiver students using pre- and post-test data. As a second step, I sketched the module-specific differences in TRC and MRC in the caregiver students after the training in order to answer research question Q2.

The increase in the median from pre- to post-test underlines the shift in competencies in the caregiver students. The decrease in the IQR and therefore the decrease in dispersion indicates that the group became more homogeneous with regard to their TRC and MRC. Analysis of the results shows a significant increase in competencies after training in both TRC and MRC. One caregiver student (C10) showed a decrease in competencies post-training; however, she had already scored very highly in the pre-test compared with the rest of the group (97/108 points). Also in the post-test, this student ranked among the best in the group (91 points). The same applied to another caregiver student (C12), who obtained the same number of points (85 points) before and after the training.

It can be assumed that the inclusion of the orphanage’s management level from the beginning played an important role in the high levels of competency increase achieved. This reality is also confirmed by Taneja *et al*. (2002) as well as Groark and McCall (2011). Moreover, group training programmes have a huge peer-learning effect when directly implemented in a social setting, for example in an orphanage. They also allow the caregivers to implement the lessons learned immediately. This is consistent with other findings (Groark & McCall, 2011). The intrinsic motivation should also not be underestimated. Proof of the intrinsic motivation of the participating caregiver students could be seen in their ongoing commitment over the course of training and in their multiple oral assurances highlighting the importance of the training.

The module-specific analysis of TRC and MRC indicates that the shift in competencies depended on the topic. For instance, in the innate intersubjectivity module, the caregiver students increased their knowledge significantly in TRC but not in MRC. Contrary to this, in the respectful caretaking module, MRC increased significantly but not TRC. In the child language development module there was no significant difference between the before and after training data. Finally, in the communicative musicality and play module, caregiver students showed a significant increase in both TRC and MRC.

The difference in competency shifts may be the result of the different trainers in the different modules. The four thematic blocks were taught by different groups of students and consequently by different lecturers. Their impact on the mediation of the content plays a crucial role. This agrees with the findings of Taneja *et al*. (2002), who also emphasise the role of the lecturer in the success of training programmes. In the current case, some of the topics were taught using a more theoretical approach (e.g. innate intersubjectivity) than others (e.g. respectful caretaking).

As result of the training, it was possible to record changes in early childhood education standards at the orphanage: caregiver students showed greater knowledge about the communicative abilities of babies and how to interact with them at the time of the post-test (see Table 1, innate intersubjectivity module, topics a and b). Moreover, MRC had increased with regard to the importance of an emotionally meaningful relationship for speech and language development as well as with regard to building an emotionally meaningful relationship (see Table 1, respectful caretaking module, topics a and b). In addition, their MRC concerning different types of songs for regulating a child’s emotions increased, as well as their knowledge about different ways of expressing emotions (see Table 1, communicative musicality and play module, topics a and b).

These results are consistent with existing evidence on interventions. A study by Groark *et al*. (2005) elicited the impact of two interventions on promoting a social–emotional relationship and attachment between caregivers and children in orphanages in St. Petersburg, Russia. Firstly, they trained the caregivers to promote responsive caregiving. Secondly, they changed staffing and structures to support relationship building. The authors suggest that training the staff and implementing structural changes are effective measures that can increase socially responsive caregiving (Groark *et al*., 2005). The present study also shows the pre- and post-test behavioural changes in caregiver and child interaction. However, we refrained from implementing structural changes, as this could have been perceived as coercive interference in Tanzanian orphanage culture.

In addition, a study by Sparling *et al*. (2005) also proved the impact of educational interventions on the developmental progress of Romanian orphans. Sparling and others argue that educational intervention programmes should start as early as possible in a child’s life – preferably from birth. This idea was also integrated into the present study. Thus, the training focused on children from birth to 3 years old and particularly during the first few months of life.

## Study limitations

Being designed as an empirical study, the sample size in particular as well as the absence of a control group and the related robustness in the results need to be critically assessed.

Other studies that have also operated in orphanages indicate similar limitations (McCall *et al*., 2010; Wright *et al*., 2014). The present study shows the first stages of a culturally sensitive and participative intervention in the field of early communication and language development in Tanzania. This is in line with Chevalier and Buckles (2013), who state that science has reached a junction where each researcher has to decide between: (1) reinforcing the ivory tower by advocating vague institutional interests (i.e. trying to ‘measure’ the institutional reality despite the complexity of real practice, whereby each attempt at quantitative survey already changes the social reality), and (2) including in academic practice an obligatory focus on the needs of society by adding a qualitative element. I now share the opinion that only the latter approach is compatible with special-needs education, for which reason this study was complemented and added to by qualitative data such as the video recordings. Here the constant movement and development of the methodology becomes apparent once again.

The results have to be seen as provisional and representative only of the specific local context. Another limitation to the scope of the results lies in the lack of a control group. Because of this, I cannot attribute with absolute certainty the behavioural changes in the caregiver students to participation in the training programme. However, the complementary qualitative video analysis does support the line of argument of the quantitative data presented (see future publications).

In general, the exploratory character of the study should be stressed again; the results should serve as a starting point for further work in the field. A more in-depth methodological review with regard to participative research is essential and crucial. Babylab Hannover (Frank & Trevarthen, 2015) is currently working on the intersubjective and intercultural (inclusive) in vivo research (III-R) approach. This approach explicitly connects the developmental psychological concept of innate intersubjectivity with the demands of inclusive work in an intercultural context. As part of the process, participative methods will be developed collaboratively with all involved participants (Frank & Trevarthen, 2015).

## Conclusion

In addition to identifying the shift in TRC and MRC in the language-learning interactions of caregiver students pre- and post-training (Q1), this sub-study also presented an analysis of module-specific differences in the acquisition of competencies (Q2). The intervention training was modelled around the culturally sensitive adaptation of the concept of RCT as a support to language development. The competencies acquired by caregiver students were then assessed using a mixture of quantitative and qualitative data whereby only the quantitative part was presented here. The results indicate some special requirements for and attributes of didactical and methodological language concepts for the further professionalisation of educators in early education and especially those working with OVC. The implications for training concepts in OVC institutional care include the following.

### Culturally sensitive feedback loops

Advanced training in early education, and especially in early relational communication as a support to language development for OVC, should address real problems in the field and allow individual designs to account for local specificities. Therefore, it is highly advisable to design the training and evaluation of the intervention in a participative way, taking into account the perspectives of all participants. This is in accordance with the findings of Wright *et al*. (2014). The training programme presented in this article is one example of how to proceed with the training of staff working in early education using the concept of RCT. As such, it can only account for the specific local context described. Transferring the concept to another setting would require yet another culturally sensitive adaptation of the concept.

### The transfer of theory into practice

In addition to the culturally sensitive feedback loops, it is crucial to establish a connection between theoretical input and the practical context encountered by early childhood professionals. This guarantees not only that theoretical knowledge (TRC) is accumulated, but also that the caregiver has the opportunity to apply the lessons learned in practice (MRC and DRC). Furthermore, the training needs to connect the working context of the individual caregiver with the institution’s management level (Nentwig-Gesemann, Fröhlich-Gildhoff, Harms & Richter, 2011). The management level has to be constantly involved in the training (Groark & McCall, 2011) to work successfully with the early education staff. Because this approach can turn out to be very resource-intensive, handouts for the management level proved useful in our context. Moreover, group training scores highly when it comes to transferring theory into practice (Nentwig-Gesemann *et al*., 2011). Additionally, the use of a video feedback system for supervision is very valuable (Digel, Olleck, Hartz & Schrader, 2010; Groark & McCall, 2011).

However, at this point these factors cannot be considered to be proven because of the exploratory nature of the study. Instead, more in-depth research is needed in the future. Future research will capture the training’s impact on the children concerned; for example in which areas does the training bring about changes in the children’s communication and language development, and to what extent? Where is it not effective? Data in this field should be used to modify and revise the training modules in both content and didactics. Furthermore, this could be followed by a longitudinal study, which would capture the long-term effects on the children (Ou, 2005). In addition, follow-up studies concerning the lasting effects of the training will be of great interest, for instance to what extent the learned topics are still meaningful for caregiver–child interaction 1 year after the training. Taneja *et al*. (2005) illustrate problems regarding sustainability in their study. Due to the high turnover of caregiver students in the orphanages, it is very difficult to firmly implant training knowledge in the institution. This underlines the need for a reliable, locally based professional who can take up the role of maintaining standards with regard to child–caregiver interaction.

This study can be considered as a first step towards the professionalisation of Tanzanian caregiver students using the adapted, culturally sensitive concept of RCT as a support to language development. An increase in the number of vulnerable children is to be expected due to the current stream of refugees to Europe (BAMF, 2016) and globally (UNHCR, 2016). For this reason, methodological considerations about culturally adapted concepts in language didactics for early childhood contexts will be of increasing importance. Sharpening the awareness of this topic was one of the main concerns of this article, as it may serve as a foundation and guide for further culturally sensitive adaptations.
